# Sestrin2 as a gatekeeper of cellular homeostasis: Physiological effects for the regulation of hypoxia‐related diseases

**DOI:** 10.1111/jcmm.16540

**Published:** 2021-05-04

**Authors:** Cunyao Pan, Zhaoli Chen, Chao Li, Tie Han, Hui Liu, Xinxing Wang

**Affiliations:** ^1^ Tianjin Institute of Environmental and Operational Medicine Tianjin China; ^2^ Department of Public Health Lanzhou University Lanzhou China

**Keywords:** cytoprotection, hypoxia, regulatory mechanism, sestrin2, therapeutic target

## Abstract

Sestrin2 (SESN2) is a conserved stress‐inducible protein (also known as hypoxia‐inducible gene 95 (HI95)) that is induced under hypoxic conditions. SESN2 represses the production of reactive oxygen species (ROS) and provides cytoprotection against various noxious stimuli, including hypoxia, oxidative stress, endoplasmic reticulum (ER) stress and DNA damage. In recent years, the determination of the regulation and signalling mechanisms of SESN2 has increased our understanding of its role in the hypoxic response. SESN2 has well‐documented roles in hypoxia‐related diseases, making it a potential target for diagnosis and treatment. This review discusses the regulatory mechanisms of SESN2 and highlights the significance of SESN2 as a biomarker and therapeutic target in hypoxia‐related diseases, such as cancer, respiratory‐related diseases, cardiovascular diseases and cerebrovascular diseases.

## 1 INTRODUCTION

Hypoxic injury is a complex pathophysiological process involving a variety of factors. Acute hypoxia can cause pulmonary arterial hypertension, decrease in myocardial contractility and arrhythmia.^1^ Lack of oxygen and blood supply to the brain can cause hypoxic‐ischaemic encephalopathy (HIE), which results in severe brain damage, and poses a significant threat to learning and memory functions.^2^ Similarly, myocardial ischaemia injury occurs when the heart suffers from hypoxia and insufficient blood supply. Moreover, hypoxia is also a feature of the cancer microenvironment. Hypoxia‐inducible factor‐1α (HIF‐1α) is often overexpressed and accumulates in cancer cells, in which HIF‐1α‐mediated signalling is a crucial pathway that regulates the metabolism and growth of solid tumours.^3^ Collectively, hypoxia causes abnormal changes in cell metabolism, function and morphology.

Sestrin2 (SESN2) belongs to the evolutionarily conserved Sestrin family and is also known as a hypoxia‐induced 95 gene (HI95).^4^ Many studies have demonstrated that SESN2 is a stress‐inducible protein that responds to various insults, such as hypoxia, energy deficiency, genotoxic stress and oxidative stress.^5^ A recent study indicated that SESN2 silencing could suppress mitochondrial biogenesis, reduce mitochondrial biological activity, and ultimately, aggravate hypoxic injury.^6^ Animal model experiments indicated that overexpression of SESN2 could improve hypoxia‐ischaemia injury.^5^, ^7^, ^8^, ^9^, ^10^, ^11^, ^12^, ^13^ SESN2 is regulated by HIF‐1α and is strongly associated with the oncogenesis and prognosis of solid tumours.^3^ Therefore, we believe that SESN2 might serve as a potential target to protect against hypoxia injury.

In this review, we summarize the latest advances regarding the compensatory mechanisms of SESN2 in hypoxia metabolism and the possible signalling pathways involved. First, we review the general background of SESN2. Subsequently, we discuss the common signalling pathways of SESN2. Finally, we consider the role of SESN2 in several hypoxia‐related diseases. This review provides novel insights into the stress modulation of SESN2 against hypoxia.

## 2 GENERAL BACKGROUND OF SESN2

In 2002, Budanov et al^4^ used cDNA microarray hybridization in an attempt to identify novel genes participating in cellular responses to prolonged hypoxia. They named a novel gene, *Hi95* (*SESN2*), whose protein product shared significant homology with a p53‐regulated GADD family member PA26. In 2008, they revealed that SESN2 provided an essential link between genotoxic stress, p53 and the mammalian target of rapamycin (mTOR) signalling pathway.^14^ Their study demonstrated that SESN2, as a target gene of P53, could activate the AMP‐responsive protein kinase (AMPK) and inhibit mTOR. Subsequently, studies have confirmed that SESN2 is a stress‐inducible protein that responds to various insults, such as hypoxia, oxidative stress, endoplasmic reticulum (ER) stress and DNA damage.^8^, ^12^, ^15^ In addition, SESN2 plays a crucial role in cancer, metabolic disorders, cardiovascular diseases and neurodegenerative disorders.^10^, ^13^, ^16^, ^17^

Therefore, it is imperative to understand the functions of SESN2 in the modulation of its associated pathophysiological mechanisms and determine how SESN2 regulates hypoxia‐related diseases.

## 3 SESN2 AND SIGNALLING PATHWAYS

### 3.1 Upstream regulators

#### 3.1.1 Hypoxia

Sestrin2 (SESN2) was first isolated as a gene that was activated in human neuroblastoma cells under hypoxia and was identified as a p53‐dependent gene.^4^ Subsequent studies found that SESN2 could be activated by energy deprivation secondary to prolonged hypoxia.^18^ HIF‐1 is a primary transcriptional regulator of cellular responses to hypoxia and has been shown to increase the expression of SESN2 in mouse epithelial tracheal cells exposed to oxidative stress.^19^

**FIGURE 1:**
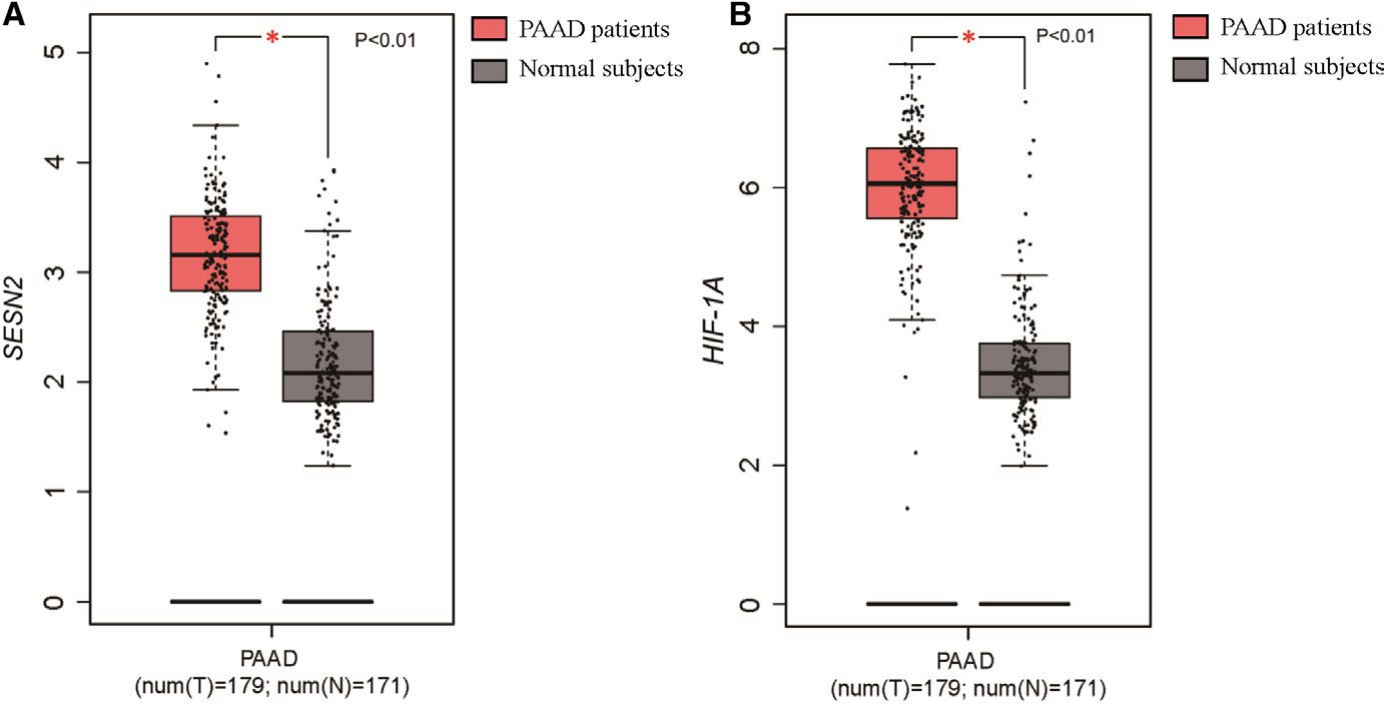
The expression of *SESN2* and *HIF1A* in PAAD (GEPIA dataset, http://gepia.cancer‐pku.cn/). *SESN2* and *HIF1A* levels were higher in patients with PAAD than that in normal samples. A, The expression of *SESN2* in PAAD. B, The expression of *HIF1A* in PAAD

**FIGURE 2:**
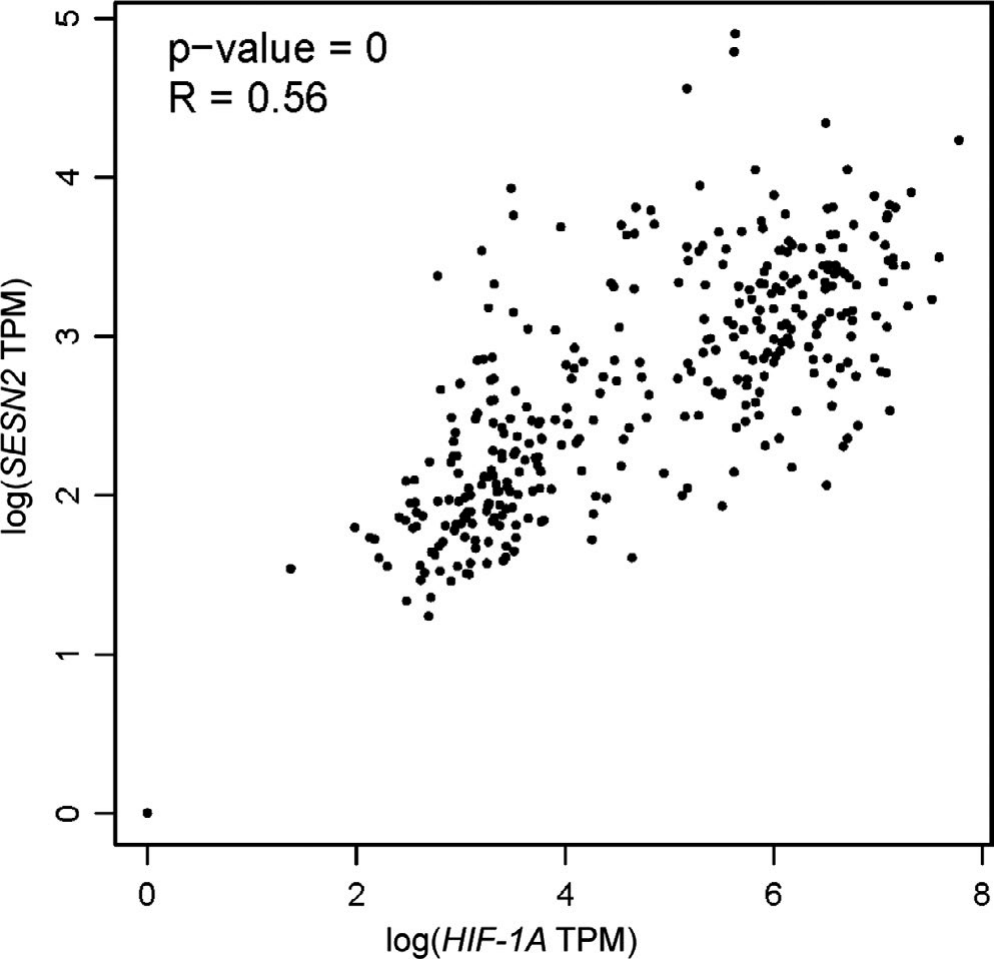
The correlation between *HIF1A* and *SESN2* in PAAD *HIF1A* was positively associated with *SESN2* (R = 0.56, *P* < 0.01)

When the environment of a cell is oxygenated, HIF‐1α can be degraded rapidly by prolyl hydroxylases (PHDs). Under hypoxic conditions, the activity of PHDs is impaired, which results in stabilization of HIF‐1α.^20^ Seo et al^21^ have indicated that AMPK is involved in HIF‐1α inhibition as a downstream signalling molecule of SESN2. Their experiments showed that overexpression of SESN2 inhibited HIF‐1α accumulation, while knockdown of AMPK reversed the HIF‐1α inhibition induced by SESN2. The activation of PHDs mediated by AMPK is responsible for the degradation of HIF‐1α. Therefore, SESN2‐AMPK signal pathway increases the degradation of HIF‐1α by regulating the activity of PHDs. Besides, HIF‐1α is often overexpressed and accumulated in cancer cells, regulating the growth of solid tumours, such as pancreatic adenocarcinoma (PDAC or PAAD).^3^ Using a gene expression profiling interactive analysis (GEPIA) data set (http://gepia.cancer‐pku.cn/), we found that the expression levels of *HIF1A* and *SESN2* were higher in PAAD than in normal samples (Figure 1A,B), intriguingly, *HIF1A* was positively associated with SESN2 (Figure 2). Since HIF‐1α is mainly regulated post‐transcriptionally, the positive associations between HIF‐1α and SESN2 may not be important. Furthermore, Shi et al^11^ studied the level of SESN2 expression in both severe and moderate hypoxic‐ischaemic (HI) rat models. The data suggested that SESN2 was activated in severe HI, but not in moderate HI. Therefore, hypoxia can induce SESN2 expression. Although the mechanisms of SESN2 induction under hypoxia remained unclear, we speculated the SESN2 and HIF‐1α interact with each other to regulate cellular metabolism.

#### 3.1.2 Oxidative stress

Oxidative stress is characterized by excessive production of reactive oxygen species (ROS) and reactive nitrogen species (RNS), resulting in cellular dysfunction. The accumulation of the products of oxidative stress causes ageing,^22^ neurodegenerative diseases,^23^ cardiovascular diseases,^24^ inflammatory response^25^ and metabolic syndrome.^26^ Recent studies demonstrated that SESN2 plays a crucial role in oxidative stress through the nuclear factor erythroid 2‐related factor 2 (NRF2) pathway.^15^, ^27^, ^28^, ^29^ The mechanism by which SESN2 activates NRF2 expression was revealed by Bae et al,^30^ who showed that the antioxidant function of SESN2 was mediated through activation of NRF2 in a manner reliant on p62‐dependent autophagic degradation of Kelch‐like ECH‐associated protein 1 (KEAP1).

Fan et al^15^ proved that SESN2 overexpression markedly decreased H_2_O_2_‐induced apoptosis and ROS generation. A study by Ro et al^31^ demonstrated that uncoupling protein 1 (Ucp1), which is localized in the mitochondrial inner membrane of mammalian brown adipose tissue (BAT) and generates heat by uncoupling oxidative phosphorylation, can be inhibited by SESN2 via a reduction in ROS accumulation. Besides, cancer cells induce ROS overexpression to support proliferation and diffusion. SESN2 can curb oxidative stress and slow tumourigenesis.^21^, ^32^ The occurrence of cancers is associated with significant downregulation of SESN2.^21^, ^33^ Therefore, SESN2 is considered a vital factor in the removal of ROS and oxidative stress regulation.

#### 3.1.3 Endoplasmic reticulum stress

The accumulation of misfolded or unfolded proteins is known as ER stress.^34^ Increasing evidence highlights the significant impact of ER stress on maintaining cellular homeostasis.^35^ It was demonstrated that SESN2 expression was increased via the protein kinase R‐like endoplasmic reticulum kinase (PERK), eukaryotic initiation factor‐2 alpha (eIF2α)/activating transcription factor‐4 (ATF4)‐dependent pathway.^36^ The unfolded protein response (UPR) is significant in oxidative stress and inflammatory responses in cancer. Ro et al^16^ suggested that SESN2 suppressed colon tumour growth through increased ER stress and p53‐mediated control over mTORC1 signalling. Bruening et al^37^ found that the upregulation of SESN2 was associated with the expression of ER stress markers ATF4, ATF3 and C/EBP‐homologous protein (CHOP) in cancer cells. Another study reported that SESN2 inhibited ER stress and inflammation through the AMPK/mTORC1 pathways.^38^

Recently, Wang et al^39^ found that *SESN2* knockdown promoted ER stress‐related cell death. Conversely, overexpressing *SESN2* in dendritic cells (DCs) decreased their apoptosis rate and inhibited ER stress‐related protein translation. Consistent with these findings, Lee et al^38^ disclosed that SESN2 suppressed impaired trophoblast invasion caused by palmitate and attenuated palmitate‐induced ER stress; conversely, knockdown of *SESN2* increased palmitate‐mediated ER stress, inflammatory signalling and apoptosis. Moreover, a recent study proved that glucose starvation caused both energy deficiency and activation of ER stress, in which SESN2 protected cells from glucose starvation‐induced cell death.^40^ The study further demonstrated that UPR‐induced SESN2 via ATF4 and NRF2 transcription factors. Taken together, SESN2 can be activated via three mechanisms: Firstly, through UPR‐induced activation of ATF4 and NRF2; secondly, by p53 in response to DNA damage; and thirdly, by the AMPK/mTORC1 pathway.

#### 3.1.4 DNA damage

DNA damage can be caused by various endogenous or exogenous stresses, including oxidative stress, oncogenic mutations and metabolic stress.^41^ It was reported SESN2, as a p53 downstream target gene, plays a crucial role in the regulation of cellular DNA damage, such as gamma or ultraviolet (UV) radiation‐induced DNA damage.^42^, ^43^ Zhao et al^44^ found that UVB‐induced SESN2 expression regulated DNA damage repair through the p53 and AKT3 pathways. Taken together, SESN2 is involved in monitoring cellular DNA damage and maintaining cellular redox homeostasis.

### 3.2 Downstream effectors

#### 3.2.1 The AMPK/mTORC1 pathway

AMP‐responsive protein kinase (AMPK), which is regulated by the AMP to ATP ratio, is considered a cellular energy sensor and plays a critical role in energy metabolism.^45^ The mechanisms underlying AMPK activation include hypoxia, glucose deprivation liver kinase B1 (LKB1) and SESN2.^46^ mTOR is present in two distinct complexes, mTORC1 and mTORC2. It was reported that inhibitors of mTOR might be available to treat cancer, cardiovascular disease, autoimmunity and metabolic disorders.^47^ AMPK, as a negative regulator of mTOR signalling, phosphorylates and inactivates mTORC1.

**FIGURE 3:**
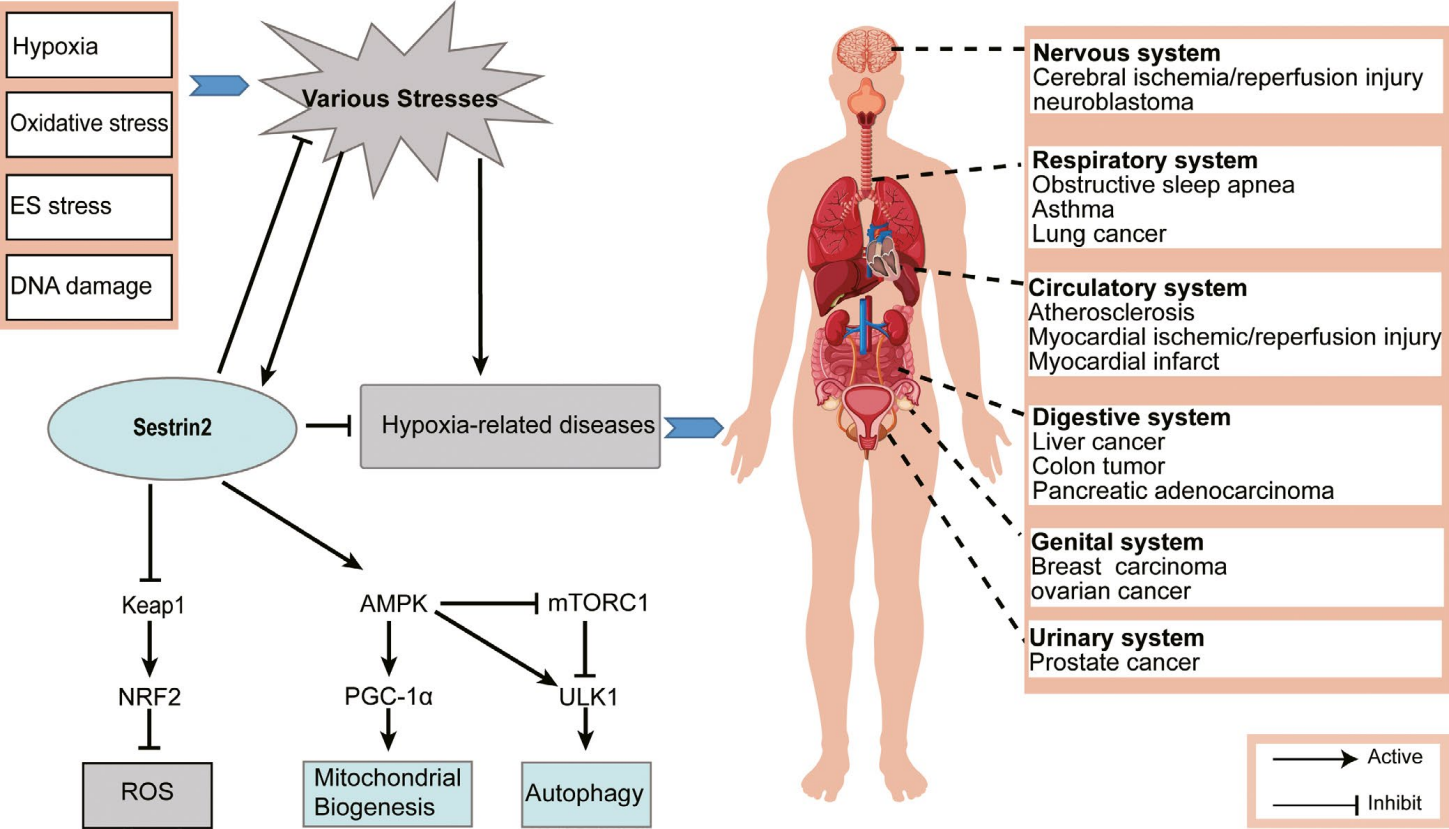
The underlying mechanism of SESN2 induction and the potential role of SESN2 as a therapeutic target in diverse systems

It is widely accepted that SESN2 inhibits mTORC1 through the activation of AMPK.^14^, ^48^ Although it remains unclear how SESN2 activates AMPK, Sanli et al^46^ presumed that SESN2 regulated AMPK activation by orchestrating the recruitment of LKB1, as well as increasing LKB1/AMPKα1β1γ1 complex expression. Peng et al^49^ demonstrated that SESN2 regulated the nutrient‐sensing Rag GTPases to control mTORC1 signalling. In addition, accumulating evidence showed that the AMPK/mTORC1 signalling pathway was significantly associated with the role of SESN2 in genotoxic stress,^14^ ROS elimination^50^ and autophagy.^51^ As previously described, SESN2, as a stress‐inducible protein, plays a crucial role in cell homeostasis and metabolism through the AMPK/mTORC1 pathway (Figure 3), and further study of this pathway will contribute to our understanding of the regulation of cellular energy metabolism.

#### 3.2.2 The KEAP1/NRF2 pathway

Nuclear factor erythroid 2‐related factor 2 (NRF2), a member of a family of basic leucine transcription factors, is involved in redox regulation, DNA repair and the prevention of apoptosis.^52^ KEAP1 functions as a sensor of oxidative stresses, as well as a negative regulator of NRF2.^53^ Under normal conditions, KEAP1 binds to NRF2 in the cytoplasm. However, under oxidative stress, NRF2 is transferred to the nucleus and binds to antioxidant response elements (AREs), thereby exerting physiological functions by activating the transcription of downstream genes.^54^ Bae et al^30^ suggested the antioxidant activity of SESN2 was mediated by NRF2 activation, which was dependent on p62‐dependent autophagy of KEAP1. Fan et al^15^ found that SESN2 played an essential role in retinal degeneration in glaucoma by enhancing NRF2 activation via KEAP1 downregulation. Moreover, obesity‐related nonalcoholic fatty liver disease could be improved via the SESN2/NRF2/haeme oxygenase 1 (HO‐1) pathway.^27^ It was reported that the KEAP1/NRF2 signalling system plays a crucial role in a range of diseases, including inflammatory diseases,^55^ stroke^56^ and cardiovascular diseases.^57^ In summary, SESN2 exerts its antioxidant defense effects via the elimination of ROS accumulation through KEAP1/NRF2 signalling activation.

#### 3.2.3 Autophagy

Autophagy is a cellular defense mechanism that is important for the maintenance of cellular homeostasis.^58^ As described earlier, SESN2 regulates the AMPK/mTORC1 signalling pathway, and mTORC1 is a major regulator of autophagy.^58^, ^59^ In the presence of nutrients, mTORC1 is activated to inhibit the Unc‐51‐like protein kinase 1 (ULK1) complex and autophagy.^60^ mTORC1 is inhibited under nutrient deprivation conditions and ULK1 complexes can lead to the formation of autophagosomes. It was reported that SESN2 activated NRF2 by promoting p62‐dependent autophagic degradation of KEAP1^30^; therefore, we hypothesized that SESN2 is closely related to autophagy. Liang et al^61^ investigated the mechanism, and revealed that the induction of autophagy by SESN2 was regulated by the c‐Jun N‐terminal kinase (JNK) pathway. Interestingly, autophagy has dual roles in cancer. On the one hand, it can suppress tumour growth by preventing the accumulation of damaged proteins and organelles^59^, ^62^, ^63^; on the other hand, it can promote tumour cell survival under conditions of excessive accumulation of the p53‐dependent autophagy protein microtubule associated protein 1 light chain 3 alpha (LC3).^64^ Taken together, these studies suggest that SESN2 plays an integral part in autophagy; however, further research is required to determine its beneficial effects and to develop ways to inhibit its adverse effects.

## 4 SESTIN2 AND DISEASES

An increasing number of studies have confirmed the stress response impact of SESN2 on hypoxia metabolism; however, SESN2 behaves differently in various diseases. Here, we present an overview of SESN2 in hypoxia‐related diseases to provide a reference for their subsequent treatment (Figure 3).

### 4.1 Cancer

**FIGURE 4:**
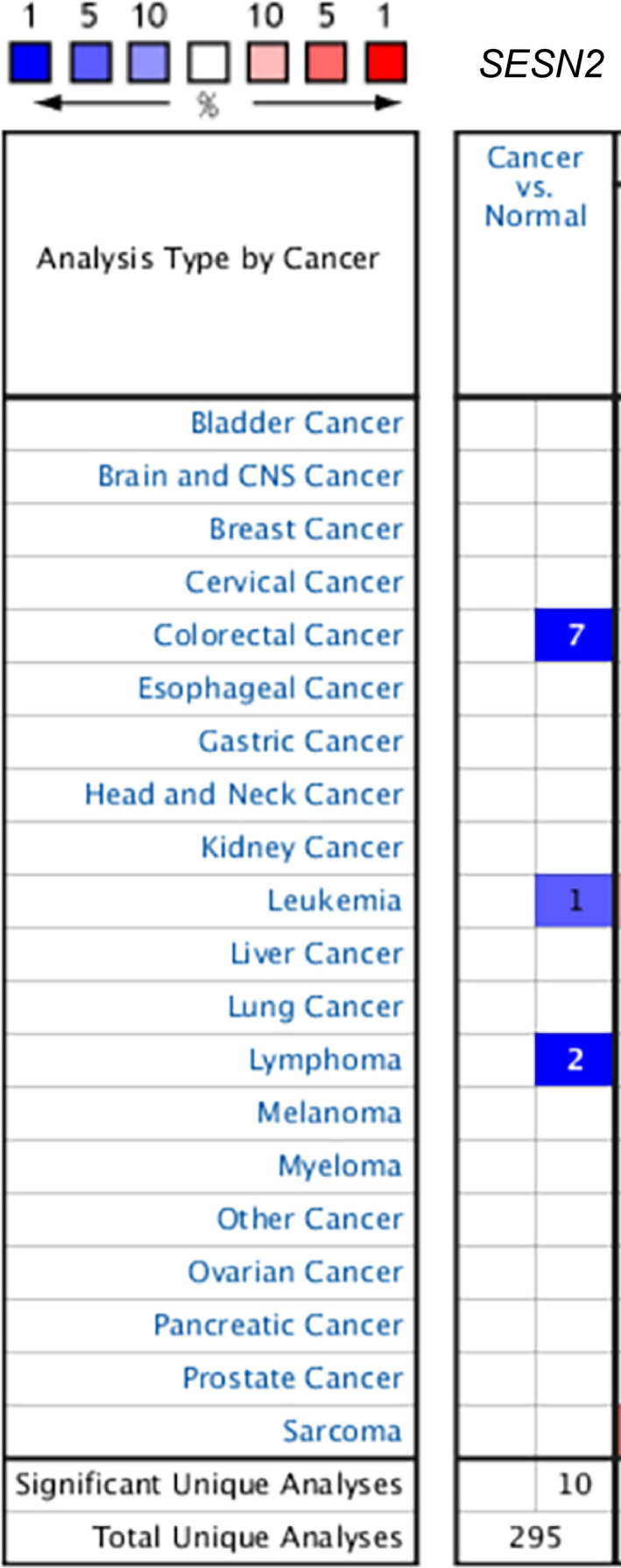
The transcription levels of *SESN2* in different types of cancers (ONCOMINE dataset, https://www.oncomine.org/). Compared with that in normal tissue, *SESN2* expression was downregulated in colorectal cancer, leukemia, and lymphoma

The occurrence of cancer is strongly associated with DNA damage, gene mutation, oxidative stress, metabolic dysregulation and inflammation.^65^ Recent research indicated that SESN2 plays a crucial role in many kinds of cancer cells. Most cancer cells show enhanced survival in the hypoxic tumour microenvironment via HIF‐1 overexpression. Several studies indicated that SESN2 levels were significantly decreased in colon adenocarcinoma tissues, and low expression of SESN2 promoted colon tumour growth.^16^ In addition, high expression of SESN2 decreased murine colon tumour cell growth both in vitro and in vivo.^63^, ^66^, ^67^ We compared the mRNA levels of *SESN2* in colon tumours with those in normal samples using the ONCOMINE (https://www.oncomine.org/) databases, which showed that the mRNA expression of *SESN2* was significantly down regulated in colon tumours (Figure 4).

The mechanism by which SESN2 regulates tumour growth was investigated by Ro et al,^16^ who proved that SESN2 was an essential mediator of p53's control over mTORC1 signalling and tumour cell growth. SESN2 inhibited colonic cell growth by suppressing both mTORC1 and ER stress. Accumulating evidence demonstrates that SESN2 can inhibit tumour growth and overcome antibiotic resistance, such as in prostate cancer,^68^ neuroblastoma^59^ and breast cancer.^69^ In non‐small cell lung cancer, high expression of SESN2 was associated with prolonged overall survival compared with that of patients with low SESN2 expression.^33^ These findings indicated that SESN2 has a tumour suppressive function. However, SESN2, as a ROS inhibitor, can play a vital role in maintaining the viability of cancer cells. Kumar et al^70^ demonstrated that SESN2 facilitated cancer cell survival under glucose starvation conditions by modulating glutamine metabolism. Dai et al showed that SESN2 induces sorafenib primary resistance in hepatocellular carcinoma.^71^ Another study conducted by Wang et al^72^ found that SESN2 overexpression weakened the tumouricidal effect of NK‐92 cells. Thus, SESN2 has both anti‐proliferative and prosurvival effects in different cancer cells. Currently, the relationship between cancer and autophagy remains debatable. Autophagy could act as a tumour suppressor or driver of cancer progression.^73^ On the one hand, early‐stage tumour cytogenesis is associated with decreased autophagy levels. On the other hand, during cancer maintenance, autophagy activity is upregulated.^74^ We speculated that the dual effects of SESN2 in cancer regulation were related to its mediated autophagy. Moreover, HIF‐1α/Bcl‐2 and adenovirus E1B 19 kD‐interacting 3(BNIP3)‐mediated mitochondrial autophagy plays a crucial role in solid tumours.^75^ However, there is no research concerning the relationship between SESN2 and the HIF‐1α/BNIP3 pathway.

In view of the dual role of SESN2 in cancer, further long‐term studies are required.

### 4.2 Respiratory related diseases

Obstructive sleep apnea (OSA), which exhibits intermittent hypoxia, could lead to arterial hypertension, stroke and other complications. These complications are associated with the triggering of oxidative stress.^76^ Bai et al^77^ demonstrated that the level of urinary SESN2 in patients with OSA was significantly higher than that of the control group and increased with the severity of OSA. Thus, the level of urinary SESN2 might be a biomarker of OSA severity. Moreover, it was reported that oxidative stress increased in asthma and caused airway inflammation and airway remodelling.^78^ Kang et al^79^ demonstrated that patients with asthma had a significantly higher plasma SESN2 level than the control group. Although the underlying mechanism for the significant upregulation of SESN2 has not been determined in patients with OSA and asthma, SESN2 might be a promising biomarker for OSA and asthma. Further research should be conducted to clarify its specificity and sensitivity.

### 4.3 Cerebral ischaemic diseases

Hypoxic‐ischaemic encephalopathy (HIE) is the leading cause of morbidity and mortality in infants, and remains the primary cause of perinatal brain injury, resulting in varying degrees of disability.^80^, ^81^ It was reported that activation of the NRF2 and AMPK pathways were important in the treatment of cerebral ischaemia.^12^, ^13^ Wang et al^13^ suggested that SESN2 promotes cerebral angiogenesis after ischaemia through the NRF2/HO‐1 pathway. Furthermore, it was reported that SESN2 overexpression decreased the brain infarct volume and diminished neuronal injury. Shi et al^12^ proved that SESN2 had a substantial neuroprotective effects after HIE via the AMPK/mTOR pathway. Stabilization of the blood‐brain barrier plays a crucial role after HI, and recombinant human (rh)‐sestrin2 attenuated blood‐brain barrier permeability and alleviated brain infarct and oedema by upregulating endogenous SESN2 levels.^11^ Chuang et al^10^ revealed that SESN2 protected hippocampal CA1 neurons against transient global ischaemia (TGI)‐induced apoptosis by regulating the phosphorylation of ribosomal protein S6 in rats.

Cerebral ischaemia/reperfusion injury (I/R) is a very complex pathophysiological process, which involves an overload of intracellular calcium (Ca), lipid peroxidation, oxygen free radical damage, apoptosis gene activation and inflammatory cytokine damage.^82^, ^83^, ^84^ SESN2 is closely associated with these processes, especially oxygen free radical damage. A study by Du et al^85^ demonstrated that SESN2 alleviated oxygen‐glucose deprivation and reoxygenation‐induced apoptosis via the NRF2 pathway. Besides, *SESN2* overexpression markedly decreased cerebral I/R injury by upregulating NRF2 in the nucleus.^86^ Conversely, *SESN2* silencing exacerbated cerebral I/R injury through the AMPK/PPARG coactivator 1 alpha (PGC1‐α) pathway.^5^

In summary, SESN2 cannot only reduce HIE and IR damage, but also significantly improves neurological function by activating various signalling pathways.

### 4.4 Ischaemic heart diseases

Coronary atherosclerotic heart diseases, such as myocardial infarction and ischaemic stroke, are major causes of morbidity and mortality worldwide.^87^ Oxidative stress is a major player in cardiac pathophysiology; therefore, it was speculated that SESN2 would be protective against cardiomyopathies. With ageing, the level of SESN2 in the heart declined, leading to an impaired AMPK/PGC‐1α signalling cascade and aggravated ischaemic insults.^88^ Yang et al^89^ found that SESN2 suppressed the activated macrophage‐mediated inflammatory response in MI via the inhibition of mTORC1 signalling. In the drosophila heart, loss of dSesn resulted in cardiac malfunction.^50^ Similar reports suggested that SESN2 exerts heart‐protective effects in ischaemic heart disease.^7^, ^8^ However, it was reported SESN2 concentrations were increased in patients with chronic heart failure (CHF), and correlated positively with the severity of CHF; indeed, augmented SESN2 levels increased the occurrence of major adverse cardiac events significantly, suggesting poor outcome in patients with CHF.^90^ According to the above studies, we speculated that SESN2 levels increased in a compensatory manner in response to oxidative stress in ischaemic heart disease. It appeared that a protective effect occurred when the SESN2 concentration was sufficient to cope with oxidative stress; however, the increase was not sufficient to counter it, which might have led to a false impression.

Although reperfusion treatment of the ischaemic myocardium was established as a highly beneficial therapy for MI, more severe injury might occur after the onset of reperfusion, which is called ischaemia‐reperfusion injury (IR injury).^91^ It was reported that pyruvate dehydrogenase (PDH), which can modulate the interaction between SESN2 and LKB1, could regulate energy metabolism to alleviate the cardiac damage caused by I/R injury.^92^ Quan et al^9^ also proved that SESN2 mediated AMPK activation to alleviate I/R injury.

Taken together, SESN2 may represent a biomarker of the extent of CHF and a novel target for the amelioration of cardiovascular diseases.

## 5 DISCUSSION

Data from various animal experimental models and clinical research indicate that SESN2 is a promising target for the treatment of hypoxia‐related diseases in humans. In this review, we briefly discussed the upstream and downstream regulators of SESN2 (Figure 3). Regulated by P53 and HIF‐1α, SESN2 is an important regulator of metabolism and oxidative stress. SESN2 can diminish ROS accumulation and activate autophagy, thus suppressing cancer, respiratory‐related diseases, cardiovascular diseases and cerebrovascular diseases (Figure 3). Although the advantageous or disadvantageous roles of SESN2 appear in different diseases, SESN2 provides a novel therapeutic target for the prevention of hypoxia‐related diseases and metabolic disorders. Further research is needed to determine the specific underlying mechanisms by which SESN2 exerts its functions.

## CONFLICT OF INTEREST

The authors declare no conflict of interest.

## AUTHOR CONTRIBUTION

**Cunyao Pan:** Conceptualization (lead); Visualization (lead); Writing‐review & editing (lead). **Zhaoli Chen:** Writing‐review & editing (supporting). **Chao Li:** Writing‐review & editing (supporting). **Tie Han:** Supervision (lead). **Hui Liu:** Visualization (supporting). **Xinxing Wang:** Project administration (lead); Writing‐review & editing (lead).
